# Trajectories of COVID-19 pandemic-related depressive symptoms and potential predictors: the FinnBrain Birth Cohort Study

**DOI:** 10.1007/s00127-023-02559-0

**Published:** 2023-09-05

**Authors:** Ru Li, Max Karukivi, Jallu Lindblom, Riikka Korja, Linnea Karlsson, Hasse Karlsson, Saara Nolvi

**Affiliations:** 1 Department of Psychiatry, University of Turku and Turku University Hospital, Medisiina A, Kiinamyllynkatu 8-10, Turku, Finland; 2 FinnBrain Birth Cohort Study, Department of Clinical Medicine, University of Turku, Turku, Finland; 3 Faculty of Social Sciences, Tampere University, Tampere, Finland; 4 Centre for Population Health Research, University of Turku and Turku University Hospital, Turku, Finland; 5 Turku Institute for Advanced Studies, University of Turku, Turku, Finland; 6 Department of Medical Psychology, Charité Universitätsmedizin, Berlin, Germany; 7 Department of Psychology and Speech-Language Pathology, University of Turku, Turku, Finland; 8 Department of Adolescent Psychiatry, University of Turku and Turku University Hospital, Turku, Finland; 9 Department of Pediatrics and Adolescent Medicine, University of Turku and Turku University Hospital, Turku, Finland

**Keywords:** Depressive symptoms, Adult temperament, Alexithymia, Coping, COVID-19

## Abstract

**Purpose:**

In the context of the COVID-19 pandemic, mental health problems have been reported, and parents of young children may be more vulnerable to psychological distress due to increased caregiving responsibilities. However, research on the heterogeneity of the longitudinal course of psychological symptoms during the pandemic and the predispositions linked with these courses is still scarce. This study aimed to identify differential trajectories of depressive symptoms among the parents of young children and investigate the role of temperament traits, alexithymia, and coping styles in the heterogeneity of the symptom trajectories.

**Methods:**

The sample consists of 844 parents from the FinnBrain Birth Cohort Study. Latent growth mixture modeling was utilized to identify trajectories of depressive symptoms from pre-pandemic between 2014 and 2019 (T0, the closest available measurement was used) to May/June 2020 (T1) and December 2020 (T2) during the pandemic. Multinomial logistic regression was used to examine temperament, alexithymia, and coping as predictors of symptom trajectories, controlling for various background factors.

**Results:**

Four trajectories of depressive symptoms were identified. Most parents experienced low and stable depressive symptoms. Negative affect, effortful control, alexithymia, emotion-diverting coping (self-distraction and venting), and avoidant coping (denial and behavioral disengagement) were predictors for subclinical stable depressive symptoms. Constructive coping (positive reframing, acceptance, and humor) protected the cohort parents from increasing or moderately high depressive symptoms.

**Conclusions:**

The findings have implications for identifying vulnerable individuals with specific traits and strengthening of constructive coping strategies as possible foci in interventions for depression during global crises.

**Supplementary Information:**

The online version contains supplementary material available at 10.1007/s00127-023-02559-0.

## Introduction

The rapid worldwide spread of the coronavirus disease (COVID-19) has had a widespread impact on mental health [1, 2]. In addition to health-related factors, such as worries about personal physical health and fear of infecting family members, restriction-related factors, such as social isolation, economic burden, and decreases in the quality of personal relationships are reportedly significant stressors contributing to psychological distress during the COVID-19 pandemic [3–5].

In the context of COVID-19, general mental health is found to have deteriorated compared with pre-pandemic period [6, 7]. Specifically, there are several longitudinal studies comparing depressive symptoms, comprising feelings of low mood, sadness, hopelessness, helplessness, inappropriate guilt, and reduced energy or fatigue [8], in pre-pandemic and pandemic periods. A study in Italy showed that pre-pandemic depressive symptoms increased during lockdown [9]. However, a study from China indicated that depression levels did not change significantly from the initial outbreak to the peak of the pandemic [10]. In a study with a German sample, no increases in psychopathological symptoms were either found, although rising symptoms were observed in a high-stress group [11]. Thus, the mixed results may be attributable to the sample characteristics. Recent meta-analysis of longitudinal studies suggested an overall small mental health influence of the pandemic but specific individuals such as females and younger adults at greater risk of mental health problems, highlighting a considerable degree of heterogeneity across populations [12–16].

Considering that the pandemic-related environmental change may be a challenge for specific populations with risk factors, it is relevant to identify heterogeneity of mental health trajectories during the pandemic. The heterogeneity refers to differential mental health outcomes, which may be caused by diverse conditions. For example, a prior study indicated that most of the participants experienced low-consistent depressive symptoms, whereas some experienced high-increasing symptoms due to differential caregiving burden and coping strategies [17].

However, to screen and prevent poor mental well-being in the long term, it is important to study on resilience and risk factors related to longitudinal response to the pandemic. Recent studies have reported certain demographics such as female gender and parenthood to be risk factors of heightened depression in the beginning of the COVID-19 pandemic [7, 18]. However, little is known about trait-like features that may be relevant to dealing with the pandemic longitudinally. Specific temperament or personality traits are related to psychological responses by influencing stress appraisal and coping strategies employed when facing stressful situations [19]. Traits such as depressive and cyclothymic temperament typically linked with high negative affect or neuroticism may be predictors for moderate-to-severe psychological distress during the COVID-19 outbreak [20]. In addition, alexithymia, a personality trait involving difficulties in identifying and expressing feelings, externally oriented style of thinking and a scarcity of imagination [21], may also contribute to pandemic-related psychological response [22, 23].

On the other hand, specific coping strategies are plausible contributors to emotional response to the pandemic. Coping processes refer to behavioral and cognitive efforts to reduce the internal and external demands that exceed personal resources [24]. Folkman et al. claimed that different types of coping may cause distinct emotional outcomes [25]. For instance, task-oriented coping methods such as acceptance and positive reframing are regarded as active or adaptive, which typically protect individuals from psychological distress under a stressful situation, whereas emotion-oriented and avoidance-oriented coping as passive or maladaptive methods tend to be related to poorer outcome in mental health [26–28]. Active coping styles have been suggested as protectors and passive coping as risk factors for depression during the pandemic [29].

Investigating potential predisposition to pandemic-related mental health is especially relevant among parents with young children, as they may be more vulnerable to psychological distress during the pandemic due to increased caregiving responsibilities [7, 18] and weaker access to social support and resources [30, 31]. Moreover, parental well-being may have spillover effects on parenting and ultimately on child well-being and longitudinal development [32, 33]. Nevertheless, there is still little knowledge on how temperament, alexithymic traits, and coping strategies influence the longitudinal patterns of pandemic-related depressive symptoms.

The present study aimed to explore unobserved subpopulations with distinct longitudinal trajectories of depressive symptoms from pre-pandemic to the first year of pandemic among Finnish parents and to investigate the potential predictors. As mental health may slightly change in general population with high heterogeneity across individuals [12–16], it was hypothesized that differential trajectories would be identified, with one trajectory being low and stable, and others characterizing larger changes in symptoms, but specific hypotheses were not set because of lacking evidence on this topic. Furthermore, based on previous research, individuals with specific traits such as high negative affect (a temperament trait linked with neuroticism) and high alexithymia were expected to have higher levels and/or more increases of depressive symptoms [34, 35]. In addition, prior research suggested active coping styles to be protective factors from and passive coping as risk factors for depression [28, 29, 36]. We, therefore, expected acceptance and positive reframing to protect the parents from depressive symptoms, and denial and behavioral disengagement to predict higher or increasing symptoms across follow-up.

## Methods

### Study participants and design

**Fig. 1:**
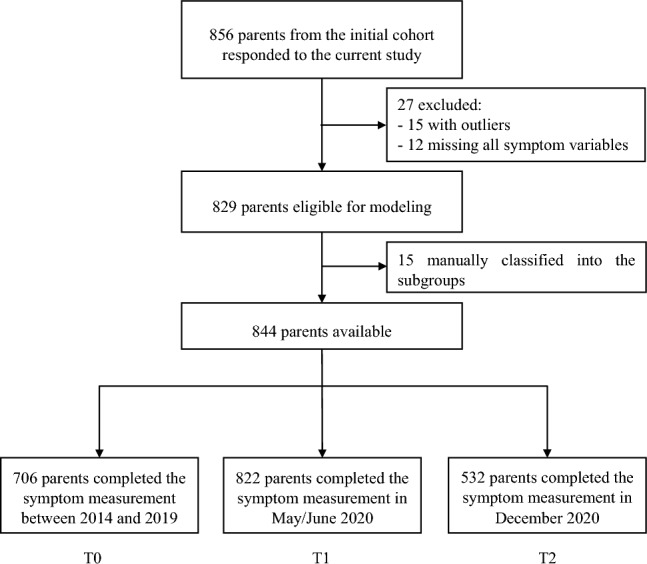
Flow chart of the enrollment and follow-up of study participants

This study is based on the FinnBrain Birth Cohort Study (*N* = 3808 families), a prospective cohort study exploring the impacts of prenatal and early life stress on child brain development and health [37]. The families were recruited in Finland between December 2011 and April 2015 from maternal welfare clinics in the South-Western Hospital District and the Åland Islands. All the participants in the cohort were invited to respond to the COVID-19 follow-up questionnaire between May and June 2020 (T1) and in December 2020 (T2); that is, around 3 months and 9 months after the first COVID-19 positive case was identified in Finland. Overall, 856 parents from the cohort responded to the current pandemic sub-study and the final study sample consists of 844 parents (see flow chart in Fig. 1, sample recruitment and attrition analyses in the Supplemental Material).

### Procedures

Demographic information about age, gender, education divided into three classes (1 = Low: High school or lower, 2 = Mid: Vocational tertiary degree, 3 = High: University degree), economic satisfaction ranging from 0 to 10 (0 = low satisfaction, 10 = high satisfaction) was collected in the first trimester of pregnancy. Alexithymia was measured at 6 months after delivery (between 2012 and 2015). Temperament was measured when their child was 12 months (between 2013 and 2016). Baseline depressive symptoms were measured at 2 or 4 year postpartum between 2014 and 2019 (T0, the closest available measurement was used). During the pandemic, the participants responded to the follow-up questionnaires electronically through Research Electronic Data Capture (REDCap) platform [38]. The T1 data included depressive symptoms, coping strategies, number of children at home (1 = one child, 2 = two children, 3 = more than two children), pandemic-related stressors, and negative life events during the past year. The T2 data included depressive symptoms, pandemic-related stressors, negative life events during the previous months, and information about remote work (1 = less than 50% time, 2 = 50% or more than 50% time).

### Measures

#### Depressive symptoms

Depressive symptoms were measured using the Edinburgh Postnatal Depression Scale (EPDS) [39]. The EPDS is a widely used 10-item self-report questionnaire applied to screen postnatal depressive symptoms with high validity and sensitivity among both mothers and fathers [40]. In this study, the measure was used because of its wide validity in different populations, and since the cohort has longitudinally employed this measure, allowing the comparison between pre-pandemic and pandemic periods. Each question is scored from 0 to 3, so the total score ranges from 0 to 30 points with higher score indicating more depressive symptoms. The scores above cut-off points of 9/10 and 12/13 are suggested for “possible depression” and “probable depression”, respectively [39, 41]. The measure showed good internal consistency across the follow-up (Cronbach’s *α* at T0 = 0.84, at T1 = 0.85 and at T2 = 0.88).

#### Temperament traits and alexithymia

Temperament traits were assessed by a 77-item version of the Adult Temperament Questionnaire (ATQ) with a 7-point Likert scale ranging from 1 (extremely untrue of you) to 7 (extremely true of you) [42]. It is a self-report instrument developed to measure 4 temperamental dimensions including negative affect, effortful control, extraversion/surgency, and orienting sensitivity. Satisfactory internal consistency and test–retest reliability of ATQ are shown in previous research [43]. In this study, the four dimensions showed adequate to good internal consistency (Cronbach's *α* = 0.86 for negative affect, 0.80 for effortful control, 0.74 for extraversion/surgency, and 0.79 for orienting sensitivity).

Alexithymic traits were measured using the Toronto Alexithymia Scale (TAS-20), which is one of the most widely used self-report scales measuring alexithymia [44–46]. It consists of 20 items divided into 3 subscales: difficulty identifying feelings (DIF), difficulty describing feelings (DDF) and externally oriented thinking (EOT). The items are rated on a 5-point Likert scale that ranges from 1 (Strongly disagree) to 5 (Strongly agree), so a total score ranging from 20 to 100 is obtained. The TAS-20 total score was used in this study with good internal consistency (Cronbach’s *α* = 0.81).

#### Coping factors

The situational coping strategies of the participants were measured using the Brief COPE, an inventory comprising 14 subscales with 2 items for each [47]. The participants were asked “How often have you been doing these things when pandemic-related problems and worries are concerned?”. Each item is rated on a 4-point Likert scale ranging from 1 (I have not been doing this at all) to 4 (I have been doing this a lot). Only 8 subscales were used in this study, including self-distraction, denial, behavioral disengagement, venting, positive reframing, acceptance, humor, and religion.

To simplify the predictor variables, exploratory factor analyses were conducted thus yielding four factors defined as following: emotion-diverting coping (2 items: self-distraction, factor loadings: 0.83, and venting 0.81); avoidant coping (2 items: denial 0.80 and behavioral disengagement 0.77); constructive coping (3 items: positive reframing 0.61, acceptance 0.64, and humor 0.78); and religion (1 item, 0.93), which together accounted for 68.5% of total variance.

#### Pandemic-related stressors and negative life events

The COVID-19 pandemic stressors were assessed at T1 and T2 employing a questionnaire with a “yes” or “no” answer for each item based on experiences of the respondents, which is modified according to the measurement for SARS-related stressors in the study by Main et al. [27]. The questionnaire covered following events: health events related to self, family members, friends, and relatives or acquaintances, free time restrictions, and economic burden. The COVID-19 stressors used in this study were derived from averaging the sum of the stressors at T1 and T2.

In addition, 18 life events (e.g., a child starting school, moving into a new house, divorce, unemployment, and serious illness or death of a child’s grandparent) that had happened during the past year (T1) and previous months (T2) were assessed by a questionnaire with a 5-point scale on each item, of which 4 or 5 indicated a perceived negative life event. The negative life events used in this study were derived from averaging the sum of the life events at T1 and T2.

### Analysis strategy

To identify unobserved subpopulations of the study sample with different linear growth patterns of depressive symptoms over 2020, latent growth mixture modeling (LGMM) was conducted using Robust Maximum Likelihood estimation with Mplus 8 software [48]. Detailed procedures for modeling are presented in the Supplementary Material.

Other statistical analyses were conducted using IBM SPSS 25.0. Missing values of all predictors and background factors were imputed by multiple imputation producing 20 imputation data sets [49] (more detail in the Supplementary Material).

To analyze the correlations between variables, the Chi-square test was used for categorical variables and Spearman’s ρ for continuous variables, Mann–Whitney *U* tests for continuous variables and binary variables, Kruskal–Wallis tests or one-way ANOVA followed by S–N–K post-hoc tests for continuous variables and polytomous variables. The multinomial logistic regression was conducted to examine the predictive factors for the trajectory groups of depressive symptoms, controlling for gender, education, economic satisfaction, number of children at home, remote work, as well as negative life events and pandemic stressors based on the preliminary analyses. Finally, a sensitivity analysis was performed conducting the logistic regression for the groups including the manually classified participants (*N* = 15) and the results between the sets of analyses were compared.

## Results

### Descriptive statistics

Of the whole sample consisting of 844 parents, 706 parents completed the measurement of depressive symptoms at T0 (83.5%, *N* = 120 at 2 year postpartum and 586 at 4 year postpartum), 822 at T1 (97.4%) and 532 at T2 (63.1%) (Fig. 1). There were no significant differences in the background factors among the respondents at T0, T1 and T2, suggesting similar characteristics of the sample across the timepoints (Table 1). Given the results of Little's missing completely at random test (*p* = 0.088) and background factors related to the missingness showed in attrition analysis (Supplementary Material), it is plausibly assumed that data on the follow-up symptoms were missing at random.

**Table 1:** Descriptive statistics and the proportion of missing data for respondents (parents completing the symptom measurement) at each timepoint

	T0 respondents (N = 706)		T1 respondents (N = 822)		T2 respondents (N = 532)	
	N (%) or mean (SD), range	Missing %	N (%) or mean (SD), range	Missing %	N (%) or mean (SD), range	Missing %
Gender		–		–		–
Women	551 (78.0%)		645 (78.5%)		411 (77.8%)	
Men	155 (22.0%)		177 (21.5%)		118 (22.2%)	
Education		3.5%		4.1%		4.1%
Low	168 (24.7%)		204 (25.9%)		121 (23.7%)	
Mid	199 (29.2%)		235 (29.8%)		148 (29.0%)	
High	314 (46.1%)		349 (44.3%)		241 (47.3%)	
Age	32.0 (4.6), 18–50	–	31.7 (4.7), 18–50	0.1%	32.0 (4.6), 19–50	0.2%
Economic satisfaction	6.1 (2.3), 0–10	4.0%	6.1 (2.3), 0–10	4.6%	6.1 (2.3), 0–10	4.5%
Number of children at home		2.5%		2.2%		1.7%
One	125 (18.1%)		135 (16.8%)		90 (17.2%)	
Two	387 (56.0%)		452 (56.2%)		289 (55.3%)	
Three or more	179 (25.9%)		217 (27.0%)		144 (27.5%)	
Remote work		34.3%		36.3%		1.9%
< 50% time	346 (74.6%)		393 (75.0%)		388 (74.3%)	
≥ 50% time	118 (25.4%)		131 (25.0%)		134 (25.7%)	
Pandemic stressors	5.5 (2.6), 0–14	2.8%	5.5 (2.7), 0–15	1.8%	6.1 (2.4), 1–14	1.7%
Negative life events	0.4 (0.7), 0–5	3.1%	0.4 (0.7), 0–5	2.1%	0.4 (0.6), 0–3	1.7%
ATQ						
Negative affect	3.9 (0.7), 1.8–6.1	11.6%	3.9 (0.7), 1.8–6.1	20.8%	3.9 (0.7), 1.8–5.9	15.8%
Effortful control	4.7 (0.7), 2.6–6.6	11.6%	4.7 (0.7), 2.6–6.6	20.8%	4.7 (0.7), 2.6–6.6	15.8%
Extraversion/surgency	4.6 (0.7), 2.2–6.6	11.6%	4.7 (0.7), 2.2–6.6	20.8%	4.6 (0.7), 2.2–6.5	15.8%
Orienting sensitivity	4.6 (0.8), 2.1–6.6	11.9%	4.5 (0.8), 2.1–6.6	21.0%	4.5 (0.8), 2.1–6.6	16.2%
TAS-20 total	40.3 (9.4), 22–72	10.2%	40.2 (9.4), 22–72	16.9%	40.5 (9.2), 22–72	12.2%
EPDS	4.9 (4.5), 0–27	–	6.7 (4.8), 0–24	–	6.8 (5.1), 0–23	–

Education: Low: high school or lower; Mid: vocational tertiary degree; High: university degree. Economic satisfaction: from 0 to 10 (0 = low satisfaction, 10 = high satisfaction)

ATQ: Adult Temperament Questionnaire. TAS-20: 20-item Toronto Alexithymia Scale. EPDS: Edinburgh Postnatal Depression Scale

### Trajectories of depressive symptoms

**Fig. 2:**
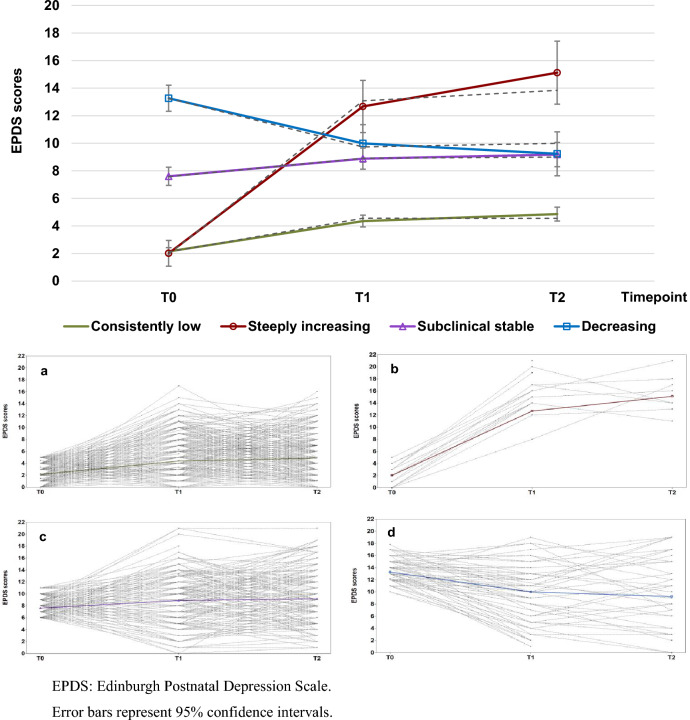
Estimated (solid line) and sample mean (dashed line) trajectories for the final four-class solution and the observed individual trajectories for each latent group. EPDS: Edinburgh Postnatal Depression Scale. Error bars represent 95% confidence intervals

The model indices for selecting optimal model are presented in Table S1 in the Supplementary Material. In brief, the AIC and BIC improved up to 5-class model, while the VLMR–LRT index suggested no significant improvement after 4-class model. Thus, the 4-class solution was adopted. The first group with a paltry increase and overall low in symptom levels across three timepoints, was named as “*Consistently low symptoms*”. The second group was named as “*Steeply increasing symptoms*”. Likewise, the third group was named as “*Subclinical stable symptoms*”, and the fourth group “*Decreasing symptoms*”. Figure 2 illustrates the estimated trajectories for the four latent groups and the observed individual trajectories for each group. The *Consistently low symptoms* (Fig. 2a), *Steeply increasing symptoms* (Fig. 2b) and *Subclinical stable symptoms* (Fig. 2c) showed relatively wide variation according to the observed individual trajectories. However, the starting points of these estimated trajectories (T0) were distinct from each other, and the standard error was small (Table 2).

**Table 2:** Estimated mean levels and slopes, and random effect parameters of the model

	Estimate	SE
*Means*		
Latent classes		
Consistently low		
Level	2.15	0.14
Slope	2.20	0.20
Steeply increasing		
Level	2.01	0.48
Slope	10.66	0.98
Subclinical stable		
Level	7.60	0.34
Slope	1.28	0.37
Decreasing		
Level	13.27	0.48
Slope	− 3.28	0.66
*Random effect parameters*		
Variances		
Level	0.87	2.27
Slope	4.55	2.12
Residual variances		
T0	2.40	2.34
T1	7.36	0.77
T2	7.68	0.98

SE: standard error

### Correlations between background information and depressive symptoms

The Spearman’s correlation matrix for continuous variables is presented in Table 3. Follow-up symptoms at T1 and T2 were related to economic satisfaction, COVID-stressors, and negative life events. In addition, according to the bivariate associations, gender, economic satisfaction, number of children at home, remote work, COVID-stressors, and negative life events were included as confounders in the further regression analyses (detailed results are described in the Supplementary Material).

**Table 3:** Spearman’s correlation matrix

	1	2	3	4	5	6	7	8	9	10	11	12	13	14	15
1. EPDS (T0)															
2. EPDS (T1)	0.49**														
3. EPDS (T2)	0.48**	0.68**													
4. Negative affect	0.27**	0.27**	0.29**												
5. Effortful control	− 0.29**	− 0.22**	− 0.24**	− 0.38**											
6. Extraversion/surgency	− 0.14**	− 0.02	− 0.03	− 0.22**	0.16**										
7. Orienting sensitivity	0.14**	0.19**	0.16**	0.19**	0.00	0.15**									
8. TAS-20	0.20**	0.11**	0.18**	0.13**	− 0.27**	− 0.25**	− 0.11**								
9. Emotion-diverting coping	0.22**	0.34**	0.28**	0.25**	− 0.10**	− 0.02	0.14**	0.01							
10. Avoidant coping	0.14**	0.19**	0.16**	0.03	− 0.13**	− 0.05	− 0.05	0.14**	− 0.04						
11. Constructive coping	− 0.08*	− 0.16**	− 0.10*	− 0.09*	0.07	0.13**	0.04	− 0.10**	− 0.03	0.03					
12. Religion	0.04	0.12**	0.07	0.07	0.00	0.05	0.12**	− 0.10**	0.00	− 0.11**	− 0.06				
13. COVID-stressors	0.10**	0.22**	0.24**	0.12**	− 0.07	0.04	0.08	− 0.04	0.17**	0.04	0.02	0.12**			
14. Negative life events	0.08*	0.21**	0.26**	0.08	− 0.08*	− 0.03	0.08*	0.06	0.07*	0.02	− 0.01	0.09*	0.16**		
15. Economic satisfaction	− 0.19**	− 0.13**	− 0.14**	− 0.09*	0.16**	0.02	− 0.07	− 0.06	− 0.03	− 0.09**	0.05	− 0.01	− 0.03	− 0.10**	
16. Age	0.00	− 0.04	− 0.02	− 0.10*	0.09*	− 0.06	− 0.02	− 0.02	− 0.07	− 0.01	0.02	0.01	− 0.01	0.02	0.04

EPDS: Edinburgh Postnatal Depression Scale; TAS-20: 20-item Toronto Alexithymia Scale

T0 = 2 or 4 year postpartum between 2014 and 2019; T1 = May and June 2020; T2 = December 2020

*p < 0.05, **p < 0.01. The pooled values after multiple imputation are presented

### Associations between temperament traits, alexithymia, and coping factors and trajectories of depressive symptoms

Alexithymia and all temperament traits, except for extraversion/surgency, were related to follow-up symptoms at T1 and T2. All the coping factors were correlated with follow-up depressive symptoms at T1 (Table 3).

All the temperament traits and alexithymia differed across the latent groups. Significant differences were found between the symptom trajectories in terms of emotion-diverting coping, avoidant coping, and constructive coping, but not in terms of religion. The *Consistently low symptoms* group presented lower emotion-diverting coping than the other groups and higher constructive coping than the *Steeply increasing symptoms* group (Table S2 in the Supplementary Material).

The results of multinomial logistic regression after controlling for the covariates are shown as odds ratio (OR) and 95% confidence interval (CI) in Table 4. There were no significant differences in the study variables between the groups of *Steeply increasing symptoms*, *Subclinical stable symptoms* and *Decreasing symptoms*. Instead, compared to the parents in the *Consistently low symptoms* group, the parents with higher negative affect were associated with higher odds for being in the group of *Subclinical stable symptoms*. Individuals with higher effortful control had lower odds of belonging to the groups of *Subclinical stable symptoms* and *Decreasing symptoms*. In addition, there was a relation between orienting sensitivity and the parents with *Decreasing symptoms*. Individuals with higher levels of alexithymia were more likely to be in the group of *Subclinical stable symptoms*. The parents using more constructive coping had lower odds for being in the groups of *Steeply increasing symptoms* and *Subclinical stable symptoms*, but those using more emotion-diverting coping and avoidant coping had higher odds of belonging to the groups of *Subclinical stable symptoms* and *Decreasing symptoms*.

**Table 4:** Multinomial logistic regression for temperament traits, alexithymia, and coping factors predicting the trajectories of depressive symptoms, controlling for the background information

Predictors	Reference consistently low (*N* = 515)					
	Steeply increasing (*N* = 26)		Subclinical stable (*N* = 229)		Decreasing (*N* = 74)	
	OR (95% CI)	*p*	OR (95% CI)	*p*	OR (95% CI)	*p*
ATQ						
Negative affect	1.30 (0.59–2.86)	0.516	**1.53 (1.07–2.19)**	**0.021**	1.14 (0.67–1.96)	0.630
Effortful control	0.73 (0.36–1.50)	0.394	**0.72 (0.53–0.98)**	**0.037**	**0.52 (0.31–0.88)**	**0.015**
Extraversion/surgency	1.13 (0.53–2.42)	0.759	0.83 (0.63–1.10)	0.199	0.71 (0.44–1.14)	0.151
Orienting sensitivity	1.26 (0.63–2.52)	0.515	1.21 (0.94–1.56)	0.141	**1.58 (1.04–2.40)**	**0.031**
TAS-20 total score	1.02 (0.97–1.07)	0.498	**1.03 (1.01–1.05)**	** < 0.001**	1.03 (1.00–1.06)	0.053
Coping factors						
Emotion-diverting	1.52 (0.93–2.50)	0.096	**1.47 (1.22–1.78)**	** < 0.001**	**1.61 (1.21–2.16)**	**0.001**
Avoidant	1.39 (0.95–2.04)	0.086	**1.38 (1.16–1.64)**	** < 0.001**	**1.37 (1.07–1.75)**	**0.014**
Constructive	**0.56 (0.36–0.87)**	**0.010**	**0.82 (0.69–0.98)**	**0.031**	0.84 (0.64–1.11)	0.225
Religion	1.12 (0.72–1.76)	0.610	1.01 (0.83–1.22)	0.952	1.28 (1.00–1.66)	0.053

Background information: gender, education, economic satisfaction, number of children at home, remote work, COVID-stressors, negative life events

ATQ: Adult Temperament Questionnaire; TAS-20: 20-item Toronto Alexithymia Scale

OR = Odds Ratio; 95% CI = 95% Confidence Interval of the OR

Statistically significant results are bolded; the pooled values after multiple imputation are presented

### Sensitivity analysis: manually classified outliers

We observed no large differences in descriptive statistics and the results of the associations between the main study variables and the symptom trajectories when the outliers were manually classified into or excluded from the latent groups (Tables S3–S5 in the Supplemental Material).

## Discussion

In this study, four trajectories of depressive symptoms were identified: “*Consistently low symptoms*” group (*N* = 515, 61.4%), “*Steeply increasing symptoms*” group (*N* = 22, 2.6%), “*Subclinical stable symptoms*” group (*N* = 229, 27.3%), and “*Decreasing symptoms*” group (*N* = 73, 8.7%). Relative to *Consistently low symptoms*, lower effortful control and higher negative affect, higher alexithymia, more use of emotion-diverting coping and avoidant coping during the pandemic had stronger associations with *Subclinical stable symptoms*. Less use of constructive coping was found to be related to *Steeply increasing symptoms* and *Subclinical stable symptoms*. Interestingly, lower effortful control and more use of emotion-diverting and avoidant coping appeared to be associated with *Decreasing symptoms* during the pandemic.

Most of the parents (61.4%) experienced *Consistently low symptoms*, similar to the findings from a previous study by Joshi et al. in which two latent groups of depressive symptoms were identified and around two-thirds of participants had low and consistent depressive symptoms during the COVID-19 pandemic [17]. A study in UK general population by Pierce et al. identified five mental health trajectories, and most of the participants had consistently good mental health during the pandemic [50]. Contextual factors such as positive attitudes towards restriction [51], and more trust in medical care and the national Government in Finland [52] may play a role in increasing the proportion of individuals with low and stable levels of symptoms.

Temperament traits were found to partially explain the heterogeneity in depressive symptoms during the pandemic. The parents with high negative affect did not experience dramatic changes in the symptoms, but they were more likely to have subclinical depressive symptoms over time. The facets of negative affect include tendency to experience and display discomfort, frustration, and sadness, which shares commonalities with the features of depressive symptoms [8, 42]. Effortful control, in turn, is a facet of top-down self-regulation defined as efficiency of executive attention, that is, the ability to activate or inhibit behavior to resolve conflicts among different responses [53, 54]. It is suggested to be a protective factor from internalizing symptoms [55, 56], which supports our finding that the higher effortful control predicted low and stable depressive symptoms in comparison with subclinical depressive symptoms. In this study, orienting sensitivity showed a link to decreasing depressive symptoms. Orienting sensitivity refers to automatic attention to both external and internal sensory events and includes general perceptual sensitivity involving the awareness of slight intensity stimulation [42, 57]. Although highly sensitive people might experience more depression related to negative stimulation [58], it was not found to be a risk trait in terms of depression during the pandemic. This may have to do with the pandemic leading to decreased external and social stimulation and consequently less stress-inducing situations for people with high orienting sensitivity.

Alexithymia has long been found to be associated with depression and post-traumatic stress symptoms [59–61]. In our study, however, neither increasing nor decreasing depressive symptoms was predicted by alexithymia, indicating that the parents with high alexithymia levels seemed not to have significant emotional responses to the pandemic. This may be to a certain degree explained by the low attention to feelings in alexithymic individuals [62]. In addition, it has been suggested that alexithymia has not directly contributed to the pandemic-related depressive symptoms but instead increased the symptom levels via interacting with perceived stress [23].

There may be item-content overlap involved in the association of specific facets of temperament and alexithymia with depressive symptoms which is likely due to the nature of personality traits being strong precursors of mental health outcomes. However, clinically, depressive symptoms (but not personality) are considered as medical outcomes, and theoretically, temperament is a core of personality, whereas depressive symptoms reflect an affective state. According to previous studies, they have been strongly suggested to be distinct constructs [63–67].

In terms of coping factors, the parents with *Subclinical stable symptoms* tended to use more emotion-diverting coping (self-distraction and venting) and avoidant coping (denial and behavioral disengagement). Individuals with high depressive symptoms may most often use dysfunctional coping strategies [68]. In line with our set of findings, these coping strategies are linked to higher depressive symptoms during the COVID-19 pandemic [69]. Neither emotion-diverting nor avoidant coping was observed to predict increasing symptoms, in contrast to the previous study [17]. The discrepant results may be due to the relatively small sample size of the group of *Steeply increasing symptoms* with high heterogeneity.

Although preexisting depressive symptoms may bring about less use of adaptive coping strategies [70], accounting for the differences between consistently low and subclinical stable depressive symptoms, the parents with *Decreasing symptoms* used constructive coping (positive reframing, acceptance, and humor) as much as those with *Consistently low symptoms*. Constructive coping was found to be the only one factor that reduced the likelihood of suffering from increasing depressive symptoms, suggesting the important protective role of constructive coping for mental health during the pandemic. This finding highlights some possible clinical implications for psychotherapeutic interventions. For instance, positive reframing is a technique involving shifting mindset and reconsidering challenging situation in a positive way, which can be trained in the traditional cognitive–behavioral therapies (CBTs). In addition, acceptance is one key technique in the third-wave CBTs, such as acceptance and commitment therapy. The findings are supported by strong clinical evidence showing that these therapies among other therapy orientations are effective in the treatment and prevention of relapse or recurrence of depression [71–73].

Interestingly, individuals lower in effortful control and using more emotion-diverting and avoidant coping were more likely to have decreasing depressive symptoms. Although the symptoms in these parents decreased from the pre-pandemic to the pandemic, their overall levels of depressive symptoms remained substantially high at the end of the follow-up. Possible explanations for the *Decreasing symptoms* are that these parents with high levels of general depressive symptoms had been poorer self-regulators and at risk in “normal lives” prior to the pandemic, and that there are unknown factors affecting the decreasing levels of depression in response to pandemic. For instance, pandemic measures may have structurally eased the distress of this group regardless of the poor coping strategies these individuals applied in response to pandemic. Further research is needed to elucidate the mechanisms behind *Decreasing symptoms*.

In the current study, depressive symptoms were measured up to December 2020, which is not the final outcome for the current pandemic. The relevance of traits and coping styles may depend on the stressor and the flexibility of the use of each strategy. For instance, avoidance can be useful with short-term uncontrollable stressors [74], and thus different association patterns may be found over time based on a more long-term observation. Therefore, further longitudinal research is needed to illustrate the role of these factors related to the long-term outcome of the pandemic.

### Strengths and limitations

To our best knowledge, this is among the first studies using longitudinal within-subject design to investigate temperament traits, alexithymia, and coping styles as potential predictors for the depressive symptom trajectories from the pre-pandemic to pandemic periods in the larger birth cohort setting.

Notwithstanding, some limitations in this study should be acknowledged. First, it is important to note that LGMM assumes continuous development over time, which may not fully capture the data characteristics in this study (e.g., the mid-measurement point indicating nonlinear trajectories). However, to a certain extent, the use of LGMM may be justified by similar trajectory patterns and superior model fit compared to alternative models like the longitudinal latent class analysis (see the Supplementary Material). Nevertheless, comparing alternative models is highly recommended for a more comprehensive understanding of symptom development and potential predictors in future research. Second, the sample size of the latent group *Steeply increasing symptoms* was relatively small compared to the other trajectory groups, for which drop-outs and the heterogeneity within the group may be the reasons. This is unfortunate as this group likely showed most clinically meaningful symptoms in the sample of the current study. Third, the majority of the participants are females, and thus the generalizability of the findings is mostly relevant to mothers of young children. To keep the sample size of each model-identified class and utilize the longitudinal cohort most effectively and ethically, all the available parents were included in the study sample that may be more representative of our initial cohort sample. In addition, considering statistical power and a parsimonious way in analyses, the sample was not split by gender. It would benefit from further studies with aims at gender differences in this regard. Fourth, temperament traits and alexithymia were measured several years earlier than the pandemic; however, they have been suggested to be highly stable traits in adult population [75, 76]. Fifth, baseline depressive symptoms (T0) were measured between 2014 and 2019, and thus the assessment of past-year negative life events did not cover the whole interval between the T0 and T1, which limits interpreting the attribution of the identified symptom trajectories to the pandemic. However, in the previous analyses of the data [2], the timepoint in which the pre-pandemic assessment was taken did not affect the results of the analyses, indicating that these timepoints can be reliably collapsed to increase the statistical power of the analysis. Sixth, the relatively high socioeconomic status of the participants indicated a low-risk population in the current study, which may limit generalizing the findings to other populations. However, as this apparently underscores the findings on the associations between predictors and the symptom trajectories, it is probable that more significant associations would be observed in high-risk populations.

## Conclusion

The present study identified four distinct longitudinal trajectories of depressive symptoms related to the pandemic. Negative affect, alexithymia, and emotion-diverting and avoidant coping were significant risk factors for subclinical depressive symptoms during the pandemic. The parents with high negative affect and alexithymic traits did not show considerable emotional responses to the pandemic. In addition, parents who had high pre-pandemic depressive symptoms with a low effortful control, high orienting sensitivity trait, and using more emotion-diverting or avoidant coping may experience *Decreasing symptoms* during the pandemic.

In contrast, effortful control and constructive coping protected individuals from increasing or moderately high levels of depressive symptoms during pandemic. These factors and especially the trainable constructive coping strategies could be possible targets for interventions focusing on adult mental health during pandemic. Furthermore, to explore long-term effects of the pandemic on the well-being of parents and children, future research on various dimensions of mental health among more diverse populations in the context of COVID-19 is warranted.

### Supplementary Information

Below is the link to the electronic supplementary material.

Supplementary file 1 (DOCX 54 KB)

## Data Availability

The data sets generated for this study will not be made publicly available because of restriction imposed by the Finnish law, and the study’s ethical permissions do not allow sharing of the data used in this study. Requests to access the data sets should be directed to the Principal investigator of the FinnBrain Birth Cohort Study (hasse.karlsson@utu.fi).
