# Supplementary material for: Trajectories of COVID-19 pandemic-related depressive symptoms and potential predictors: the FinnBrain Birth Cohort Study
Source: Soc Psychiatry Psychiatr Epidemiol. 2023 Sep 5;59(1):151–63. doi: 10.1007/s00127-023-02559-0 (PMC10799828; doi:10.1007/s00127-023-02559-0)
Supplement: Supplementary file 1 — (DOCX 54 KB) [file 127_2023_2559_MOESM1_ESM.docx]

**Trajectories of COVID-19 Pandemic-related Depressive Symptoms and Potential Predictors: The FinnBrain Birth Cohort Study**

**Attrition analysis comparing T2 responders with the initial cohort and non-responders**

In prior research, it was reported that the parents responding at T1 were older and more highly educated compared to the initial FinnBrain cohort [1]. In addition, more mothers than fathers in the cohort responded at T1 (17.6% vs. 7.1%, χ² = 146.90, *p* < .001). Similarly, the parents responding to the symptom measurement at T2 were also older (*p* < .001), had higher levels of education (*p* < .001) and higher economic satisfaction (*p* = .003) compared to non-responders in the initial cohort, while no gender differences were found. Among the participants of this study, the missing value analysis indicated that the T2 responders reported more “High” education (47.1% versus 38.3%, *p* = .039) and more COVID-19 stressors (Z = -9.24, *p* < .001) compared to the non-responders at T2. No differences were found in other background factors and baseline depressive symptoms between the responders and non-responders at T2 in the current sample. Moreover, after conducting auxiliary analysis based on the distribution of missing data across trajectory classes, we did not find significant differences in the missing values on the symptoms at T1 or T2 between the symptoms trajectories. T0 symptoms showed a difference between the trajectory groups. However, the group of *Decreasing symptoms* and *Steeply increasing symptoms* had similarly few missing data on the T0 symptoms (1 and 3, respectively), so the missingness of T0 symptoms may not actually impact the symptom trajectories. Additionally, the Little’s Missing Completely at Random Test suggested no relations between the symptoms and the missingness (*p* = .088).

Procedures for latent growth mixture modeling (LGMM)

There was also a mid-measurement point between the time points of Spring 2020 (T1) and December 2020 (T2); however, since the pandemic situation was relatively calm during the Summer 2020, and the individuals had few symptoms (and less heterogeneous response) related to the pandemic, the trajectory modeling of this time point was difficult. In the early stages of analysis, we also attempted to conduct non-linear modeling of four time points but were not successful – and also the piecewise modeling was not possible due to too few time points. Hence, T1 and T2 selected as the timepoints in the current study proved most successful to indicate the heterogeneous development of the symptoms, and the timepoint in the summer was thus left out of the final analyses.

Mplus 8 software was used to examine both longitudinal factor structures and conduct LGMM [2]. Missing data in all modeling was handled using Robust Maximum Likelihood estimation. All the measures were treated as continuous and interval-scaled. First, the factor structures of the EPDS were examined using structural equation modeling to ensure the longitudinal fit of the original factor structure. The fit of the models was evaluated using a Chi-square test, the root mean square error of approximation (RMSEA, with values close to or lower than .06 indicating a good fit), the comparative fit index (CFI, with values close to or higher .90 indicating a suitable model), and the standardized root mean square residual (SRMR, with values at or below .08 reflecting good fit) [3]. The longitudinal factor analysis of the EPDS across time points T0, T1 and T2 showed acceptable fit with the data (*χ²* [369] = 838.878, *p* < .001, CFI = 0.93, RMSEA = 0.039, SRMR = 0.049), when the errors of the consecutive items 1 and 2 were allowed to correlate.

Second, to identify unobserved subpopulations of the sample with different growth patterns of depressive symptoms over time, LGMM using Robust Maximum Likelihood estimation with starting values of 500 sets was conducted. It should be noted that a mixture model is a measurement model typically using observed variables as indicators of one or more latent variables. Based on the relatively large sample size and successful replication of the one-factor structure of the EPDS across the three time points, and to avoid too heavy model, we used the EPDS score as observed variable instead of using latent variables in the LGMM analysis. By the increasing the number of latent groups, the best‐fitting model was determined based on the following indices: log likelihood (LL) with higher values, and Akaike information criterion (AIC) and Bayesian information criterion (BIC) with lower values indicating better model fit, Entropy with values closer to 1.0 indicating higher classification accuracy, posterior probabilities of class membership, and Vuong-Lo-Mendell-Rubin likelihood ratio test (VLMR-LRT) and Bootstrapping likelihood ratio test (BLRT) for k versus k-1 groups, with *p* values lower than .05 suggesting that k groups are significantly superior compared to k-1 groups [4].

856 parents from the initial cohort responded to the current pandemic sub-study. Of which 12 parents with missing on all variables were excluded from modeling. Thus, we first intended to perform the LGMM with the sample of 844 parents but got a warning showing that the latent variable covariance matrix is not positive definite. Despite attempts to fix the variance to zero, the issue persisted. The corresponding plot revealed that the mis-specified model was unable to accurately represent the diverse trajectory patterns and failed to capture the substantial presence of high symptom scores across the time points. It was observed that there were 15 cases with extreme values (3 standard deviations above the mean). These cases were speculated as potential influencers of trajectory identification and could contribute to the mis-specification of the model. We therefore tried to exclude 15 cases with outliers from the sample, thus obtaining a sample comprising 829 parents for modeling, and this problem was addressed. Since the time between T0 and T1 was not associated with the levels of depressive symptoms at any timepoint and the symptom changes from T0 to T1, we used the average time (919 days) for modeling. The interval between T1 and T2 was 7 months, that is about 210 days. Therefore, the first interval (T0 to T1) was coded as 1, and the second (T1 to T2) was coded as 0.23 (i.e., 210 divided by 919). That is, the time coding 0.00, 1.00 and 1.23 was respectively used for T0, T1 and T2, which showed a model with non-linear trajectories, but better model fit than the equal-interval model with lower AIC (11157.09 vs. 11225.05) and BIC (11237.34 vs. 11305.29).

Importantly, the LGMM allows for differences in growth parameters across latent subgroups and relies on the assumption for the relationship between the observed outcomes and time, which may have influences on the parameter estimates and model solution. To select a model that provides better clarity in understanding the trajectories, we compared several models, including latent class growth analysis (LCGA), which assumes homogeneous growth trajectories within a class by restricting within-class variance [5], longitudinal latent class analysis (LLCA), which does not incorporate time trend information, and latent growth mixture modeling (LGMM). For both LCGA and LGMM, the AIC and BIC indices improved up to 5-class model. The significant p values of BLRT from 2- to 5-class model did not identify the best-fitting model. VLMR-LRT index suggested no significant improvement after the 4-class model. LGMM is suggested to be used when it showed a better model fit (LL: -5561.55 vs. -5583.94, BIC: 11237.34 vs. 11261.98) [6].

Similarly, as the number of latent classes increased, the AIC and BIC values of the LLCA modeling decreased, and the p-value of VLMR-LRT was significant up to the 4-class model. The model fit parameters clearly indicate that the LGMM outperformed the LLCA, with a higher loglikelihood (-5561.546 vs. -5814.210) and lower BIC (11237.336 vs. 11749.707). Furthermore, the 4-class model exhibited similar trajectory patterns to the LGMM, including two classes with relatively stable symptoms at low and high levels, as well as one increasing and one decreasing symptoms. Accordingly, the estimated time-specific residuals did not show noticeable skewness, connoting that the LGMM captured the data without significantly biased estimates of the trajectories. Thus, the 4-class solution determined by LGMM was adopted, and the model fit indices are presented in **Table S1**.

After performing the LGMM, we tested if the timing interval between T0 and T1 is different across the trajectory groups by using ANOVA. No significant differences were observed in the interval among the four latent groups identified in our study (F = .975, p = .404), suggesting that the interval should not be a major problem when interpreting the findings.

As mentioned above, 15 cases with outliers were excluded from the sample for modeling. Therefore, after the trajectory modeling, in order to keep the sample size as large as possible for subsequent analyses on the relations between the trajectories and predictors, and given that the outliers may reflect practical significance, these outliers were classified manually according to the membership probabilities produced by the model and the trajectory patterns. A few missing values were imputed by hot deck imputation based on gender, education, economic satisfaction, number of children. The results obtained after excluding the outliers are presented in **Table S3–S5**, showing almost identical results to those including the outliers.

**Multiple imputation**

The multiple imputation (MI) is applied to impute the missing values of the predictors and background variables using the IBM SPSS 25.0. MI is an inclusive method providing valid statistical inferences and commonly applicable under the assumption of missing at random (MAR) [7, 8]. Given the separate variance t-tests showing that the missing values of temperament traits and alexithymia were dependent on age, education levels, and economic satisfaction, and missing data on depressive symptoms were not related to the trajectories, MAR assumption was reasonably fulfilled. These variables as well as gender reflecting the main characteristics of the sample were empirically used as auxiliary variables in the imputation. Moreover, in longitudinal studies follow-up measuring a subsample and the use of multiple and large-scale assessment, the MAR assumption is often tenable. Hence, the follow-up data related to the pandemic including number of children at home, remote work, pandemic stressors and negative life events were also included in the imputation.

In our study, using the Markov Chain Monte Carlo (MCMC) with predictive mean matching (PMM) as the model for the scale variables, missing data were imputed 20 times as recommended for 10–30% missing information [9]. Subsequently, standard statistical analyses were conducted to investigate the study aims, and the estimates were based on the 20 MI datasets producing a pooled results with the relative efficiency for all the predictors greater than 99% in the current study.

**Correlations between background information and depressive symptoms**

The results of Kruskal-Wallis test indicated that parents in the group of Steeply increasing symptoms experienced more negative life events with a pooled value of the mean rank = 540.5, when comparing to the parents in the groups of Consistently low symptoms (mean rank = 402.2), Subclinical stable symptoms (mean rank = 431.7) and Decreasing symptoms (mean rank = 466.6). In contrast, with using One-way ANOVA, the parents of the Consistently low symptoms group reported higher economic satisfaction (Mean = 6.4, SD = 2.2) compared to the parents of Subclinical stable symptoms (Mean = 5.7, SD = 2.3) and Decreasing symptoms (Mean = 5.3, SD = 2.6) groups. Additionally, the parents with consistently low depressive symptoms had smaller number of COVID-19 stressors (Mean = 5.1, SD = 2.6) compared to those with steeply increasing symptoms (Mean = 6.4, SD = 3.1), subclinical stable symptoms (Mean = 6.0, SD = 2.7) and decreasing symptoms (Mean = 6.3, SD = 3.0).

The Chi-square tests showed significant relations between number of children at home and the symptom trajectories (χ² = 16.31, *p* = .012), with more parents in the group of Decreasing symptoms having only one child staying at home, compared to those in the group of Consistently low symptoms during the pandemic (31.3% versus 13.9%, *p* < .05). Although there were no significant differences among the trajectories in terms of gender, pairwise differences were observed between some of the latent groups, with more men in the group of Consistently low symptoms (68.1% versus 59.5% women, *p* < .05) and more women in the group of Subclinical stable symptoms (29.0% versus 21.4% of men, *p* < .05). Likewise, there were more participants who had received “High” education in the group of Consistently low symptoms (65.1% versus 54.8% “Low” education, *p* < .05) and more “Low” education in the group of Decreasing symptoms (12.0% versus 6.3% “High” education, *p* < .05). In addition, more parents who reported to have worked remotely less than half working time (62.8%) were classified in the group of Consistently low symptoms, compared to those who reported more remote work (52.9%).

**Table S1** The latent growth mixture model indices: trajectories of depressive symptoms

| Fit Indices | Number of Latent Groups | | | | |
| --- | --- | --- | --- | --- | --- |
|  | *1* | *2* | *3* | *4* | *5* |
| LL | -5678.026 | -5608.277 | -5572.625 | -5561.546 | -5554.526 |
| AIC | 11372.052 | 11238.554 | 11173.250 | 11157.093 | 11147.052 |
| BIC | 11409.814 | 11290.477 | 11239.333 | 11237.336 | 11236.736 |
| Entropy |  | 0.73 | 0.74 | 0.75 | 0.76 |
| VLMR (*p*) |  | 0.008 | <.001 | 0.007 | 0.163 |
| BLRT (*p*) |  | <.001 | <.001 | <.001 | <.001 |
| Class Size (ALCP) |  |  |  |  |  |
| *Class 1* | 829 | 657 (.94) | 61 (.93) | 67 (.90) | 75 (.86) |
| *Class 2* |  | 172 (.85) | 542 (.91) | 515 (.90) | 220 (.77) |
| *Class 3* |  |  | 226 (.79) | 19 (.72) | 8 (.81) |
| *Class 4* |  |  |  | 228 (.78) | 498 (.88) |
| *Class 5* |  |  |  |  | 28 (.71) |

LL = Loglikelihood, AIC = Akaike Information Criterion, BIC = Bayesian Information Criterion, VLMR = Vuong-Lo-Mendell-Rubin likelihood ratio test, BLRT = Bootstrapping likelihood ratio test, ALCP = Average latent class probabilities.

**Table S2** Mean values of temperament traits, alexithymia and coping factors by latent groups, and results of One-way ANOVA for group comparisons

|  | Latent Groups | | | | *F* | *p* |
| --- | --- | --- | --- | --- | --- | --- |
|  | Consistently low | Steeply increasing | Subclinical stable | Decreasing |  |  |
| ATQ |  |  |  |  |  |  |
| *Negative affect* | 3.75^a^ | 4.03^ab^ | 4.03^b^ | 4.12^b^ | 16.56 | < .001 |
| *Effortful control* | 4.82^a^ | 4.78^a^ | 4.49 | 4.36 | 14.78 | < .001 |
| *Extraversion/surgency* | 4.71 | 4.73 | 4.56 | 4.53 | 2.75 | .042 |
| *Orienting sensitivity* | 4.45 | 4.76 | 4.69 | 4.85 | 7.37 | < .001 |
| TAS-20 | 39.15 | 40.29 | 42.20 | 41.78 | 5.17 | .002 |
| Coping Factors |  |  |  |  |  |  |
| *Emotion-diverting* | -.15 | .32^a^ | .22^a^ | .29^a^ | 11.46 | < .001 |
| *Avoidant* | -.12 | .24 | .19 | .18 | 7.30 | < .001 |
| *Constructive* | .08^a^ | -.46^b^ | -.09^ab^ | -.10^ab^ | 3.73 | .011 |
| *Religion* | -.04 | .14 | -.00 | .23 | 1.74 | .157 |

ATQ: Adult Temperament Questionnaire. TAS-20: 20-item Toronto Alexithymia Scale.

S-N-K post-hoc analyses: groups sharing same superscript = no significant differences.

# Supplementary Table (Outliers Excluded)

**Table S3** Descriptive statistics on background information of the sample

|  | T0 respondents  (N = 692) | |  | T1 respondents  (N = 807) | |  | T2 respondents  (N = 523) | |
| --- | --- | --- | --- | --- | --- | --- | --- | --- |
|  | N (%) or  mean (SD), range | Missing % |  | N (%) or  mean (SD), range | Missing % |  | N (%) or  mean (SD), range | Missing % |
| Gender |  | - |  |  | - |  |  | - |
| *Women* | 538 (77.7%) |  |  | 631 (78.2%) |  |  | 405 (77.4%) |  |
| *Men* | 154 (22.3%) |  |  | 176 (21.8%) |  |  | 118 (22.6%) |  |
| Education |  | 3.3% |  |  | 4.0% |  |  | 3.8% |
| *Low* | 161 (24.1%) |  |  | 196 (25.3%) |  |  | 118 (23.5%) |  |
| *Mid* | 196 (29.3%) |  |  | 232 (29.9%) |  |  | 146 (29.0%) |  |
| *High* | 312 (46.6%) |  |  | 347 (44.8%) |  |  | 239 (47.5%) |  |
| Age | 32.1 (4.6), 18–50 | - |  | 31.7 (4.7), 18–50 | 0.1% |  | 32.0 (4.7), 19–50 | 0.2% |
| Economic satisfaction | 6.2 (2.3), 0–10 | 3.8% |  | 6.1 (2.3), 0–10 | 4.5% |  | 6.1 (2.3), 0–10 | 4.2% |
| Number of children  at home |  | 0.1% |  |  | 0.4% |  |  | - |
| *One* | 125 (18.1%) |  |  | 135 (16.8%) |  |  | 90 (17.2%) |  |
| *Two* | 387 (56.0%) |  |  | 452 (56.2%) |  |  | 289 (55.3%) |  |
| *Three or more* | 179 (25.9%) |  |  | 217 (27.0%) |  |  | 144 (27.5%) |  |
| Remote work |  | 32.9% |  |  | 35.1% |  |  | 0.2% |
| *< 50% time* | 346 (74.6%) |  |  | 393 (75.0%) |  |  | 388 (74.3%) |  |
| *≥ 50% time* | 118 (25.4%) |  |  | 131 (25.0%) |  |  | 134 (25.7%) |  |
| Pandemic stressors | 5.5 (2.6), 0–14 | 0.9% |  | 5.5 (2.7), 0–15 | - |  | 6.1 (2.4), 0.5–13.5 | - |
| Negative life events | 0.4 (0.7), 0–5 | 1.2% |  | 0.4 (0.7), 0–5 | 0.2% |  | 0.4 (0.6), 0–3 | - |
|  |  |  |  |  |  |  |  |  |
| ATQ |  |  |  |  |  |  |  |  |
| *Negative affect* | 3.9 (0.7), 1.8–6.1 | 11.4% |  | 3.9 (0.7), 1.8–6.1 | 20.8% |  | 3.9 (0.7), 1.8–5.9 | 15.9% |
| *Effortful control* | 4.7 (0.7), 2.6–6.6 | 11.4% |  | 4.7 (0.7), 2.6–6.6 | 20.8% |  | 4.7 (0.7), 3.0–6.6 | 15.9% |
| *Extraversion/surgency* | 4.6 (0.7), 2.2–6.6 | 11.4% |  | 4.7 (0.7), 2.2–6.6 | 20.8% |  | 4.6 (0.7), 2.2–6.5 | 15.9% |
| *Orienting sensitivity* | 4.6 (0.7), 2.1–6.6 | 11.7% |  | 4.5 (0.8), 2.1–6.6 | 21.1% |  | 4.5 (0.8), 2.1–6.6 | 16.3% |
| TAS-20 total | 40.2 (9.4), 22–72 | 10.3% |  | 40.1 (9.4), 22–72 | 17.0% |  | 40.5 (9.2), 22–72 | 12.2% |
| EPDS | 4.6 (4.1), 0–18 | - |  | 6.6 (4.6), 0–21 | - |  | 6.6 (5.0), 0–21 | - |

Education: Low: high school or lower; Mid: vocational tertiary degree; High: university degree. Economic satisfaction: from 0 to 10 (0 = low satisfaction, 10 = high satisfaction).

ATQ: Adult Temperament Questionnaire. TAS-20: 20-item Toronto Alexithymia Scale. EPDS: Edinburgh Postnatal Depression Scale.

**Table S4** Mean values of temperament traits, alexithymia and coping factors by latent groups, and results of One-way ANOVA for group comparisons

|  | Latent Groups | | | | *F* | *p* |
| --- | --- | --- | --- | --- | --- | --- |
|  | Consistently low | Steeply increasing | Subclinical stable | Decreasing |  |  |
| ATQ |  |  |  |  |  |  |
| *Negative affect* | 3.74^a^ | 3.90^ab^ | 4.17^b^ | 4.09^b^ | 16.557 | < .001 |
| *Effortful control* | 4.82^a^ | 4.86^a^ | 4.49 | 4.35 | 14.982 | < .001 |
| *Extraversion/surgency* | 4.71^ab^ | 4.87^a^ | 4.56^ab^ | 4.49^b^ | 3.652 | .012 |
| *Orienting sensitivity* | 4.45 | 4.77 | 4.69 | 4.80 | 6.451 | < .001 |
| TAS-20 | 39.15 | 38.84 | 42.20 | 41.86 | 5.281 | .001 |
| Coping Factors |  |  |  |  |  |  |
| *Emotion-diverting* | -.15^a^ | .34^b^ | .22^ab^ | .22^ab^ | 10.513 | < .001 |
| *Avoidant* | -.12 | .09 | .19 | .21 | 7.195 | < .001 |
| *Constructive* | .08^a^ | -.43^b^ | -.09^ab^ | -.11^ab^ | 3.366 | .018 |
| *Religion* | -.04 | .28 | -.00 | .27 | 2.521 | .057 |

ATQ: Adult Temperament Questionnaire. TAS-20: 20-item Toronto Alexithymia Scale.

S-N-K post-hoc analyses: groups sharing same superscript = no significant differences.

**Table S5** Multinomial logistic regression for temperament traits, alexithymia, and coping factors predicting the trajectories of depressive symptoms, controlling for the background information

|  | Reference Consistently low (N = 515) | | | | | |
| --- | --- | --- | --- | --- | --- | --- |
| Predictors | Steeply increasing (N = 19) | | Subclinical stable (N = 228) | | Decreasing (N = 67) | |
|  | OR (95% CI) | *p* | OR (95% CI) | *p* | OR (95% CI) | *p* |
| ATQ |  |  |  |  |  |  |
| *Negative affect* | 1.08 (.42–2.78) | .876 | **1.55 (1.08–2.22)** | **.019** | 1.21 (.68–2.15) | .520 |
| *Effortful control* | 1.12 (.48–2.62) | .799 | **.70 (.52–.96)** | **.027** | **.51 (.29–.87)** | **.015** |
| *Extraversion/Surgency* | 1.40 (.55–3.54) | .482 | .84 (.64–1.10) | .208 | .67 (.41–1.11) | .118 |
| *Orienting sensitivity* | 1.20 (.56–2.55) | .640 | 1.23 (.95–1.58) | .119 | 1.54 (1.00–2.39) | .053 |
| TAS-20 | 1.00 (.94–1.07) | .997 | **1.03 (1.02–1.05)** | **< .001** | 1.03 (1.00–1.06) | .053 |
| Coping Factors |  |  |  |  |  |  |
| *Emotion-diverting* | 1.52 (.93–2.50) | .096 | **1.47 (1.22–1.78)** | **< .001** | **1.61 (1.21–2.16)** | **.001** |
| *Avoidant* | 1.39 (.95–2.04) | .086 | **1.38 (1.16–1.64)** | **< .001** | **1.37 (1.07–1.75)** | **.014** |
| *Constructive* | **.56 (.36–.87)** | **.010** | **.82 (.69–.98)** | **.031** | .84 (.64–1.11) | .225 |
| *Religion* | 1.12 (.72–1.76) | .610 | 1.01 (.83–1.22) | .952 | 1.28 (1.00–1.66) | .053 |

Background information: gender, education, economic satisfaction, number of children at home, remote work, pandemic stressors, negative life events.

ATQ: Adult Temperament Questionnaire. TAS-20: 20-item Toronto Alexithymia Scale.

OR = Odds Ratio; 95% CI = 95% Confidence Interval of the OR.

Statistically significant results are bolded; the pooled values after multiple imputation are presented.

**References**

1. Nolvi S, Karukivi M, Korja R, Lindblom J, Karlsson L, Karlsson H (2021) Vanhempien masennus- ja ahdistusoireet lisääntyivät COVID-19-pandemian alkuvaiheessa - FinnBrain-syntymäkohorttitutkimus [Symptoms of depression and anxiety among parents increased in the early stages of the COVID-19 pandemic – FinnBrain birth cohort study]. Duodecim; Lääketieteellinen Aikakauskirja 137.

2. Muthen LK, Muthen BO. Mplus User's Guide. Eighth Edition. 1998-2017. Los Angeles, CA. Muthén & Muthén.

3. Hu L, Bentler PM (1999) Cutoff criteria for fit indexes in covariance structure analysis: Conventional criteria versus new alternatives. Structural Equation Modeling 6(1):1. https://doi.org/10.1080/10705519909540118

4. Ram N, Grimm KJ (2009) Growth Mixture Modeling: A Method for Identifying Differences in Longitudinal Change Among Unobserved Groups. Int J Behav Dev 33(6):565-576. https://doi.org/10.1177/0165025409343765

5. Jung T, Wickrama KAS (2008) An Introduction to Latent Class Growth Analysis and Growth Mixture Modeling. Social and Personality Psychology CompassSocial and Personality Psychology CompassSocial and Personality Psychology Compass 2(1):302. https://doi.org/https://doi.org/10.1111/j.1751-9004.2007.00054.x

6. Muthén B (2006) The potential of growth mixture modelling. Infant and Child DevelopmentInfant and Child DevelopmentInfant and Child Development 15(6):623. https://doi.org/https://doi.org/10.1002/icd.482

7. Mühlenbruch K, Kuxhaus O, di Giuseppe R, Boeing H, Weikert C, Schulze MB (2017) Multiple imputation was a valid approach to estimate absolute risk from a prediction model based on case-cohort data. J Clin Epidemiol 84:130-141. https://doi.org/10.1016/j.jclinepi.2016.12.019

8. Little R, Rubin D. (2019) Bayes and Multiple Imputation. In: Little R and Rubin D (ed) Statistical Analysis with Missing Data, 3rd edn. https://doi.org/10.1002/9781119482260.ch10

9. Graham JW, Olchowski AE, Gilreath TD (2007) How many imputations are really needed? Some practical clarifications of multiple imputation theory. Prev Sci 8(3):206-213. https://doi.org/10.1007/s11121-007-0070-9
